# Novel Dual DPP-IV/ACE Inhibitory Peptides from Tartary Buckwheat: From Inhibition Kinetics to Cellular Bioactivity

**DOI:** 10.3390/foods15213912

**Published:** 2026-10-31

**Authors:** Liangzhen Jiang, Shiqi Liu, Ping Kan, Yan Lv, Zhibin Lv, Dingtao Wu, Liang Zou, Dabing Xiang

**Affiliations:** 1 College of Food and Biological Engineering, Chengdu University, Chengdu 610106, Chinawudingtao@cdu.edu.cn (D.W.); dabingxiang@163.com (D.X.); 2 Key Laboratory of Coarse Cereal Processing, Ministry of Agriculture and Rural Affairs, Sichuan Provincial Engineering Research Center for Coarse Cereal Industry, Chengdu 610106, China; zouliangcdu@126.com; 3 Department of Medical Instruments and Information, College of Biomedical Engineering, Sichuan University, Chengdu 610041, China; lvzhibin@pku.edu.cn

**Keywords:** Tartary buckwheat, bifunctional peptide, DPP-IV, ACE, inhibition kinetics, endothelial protection

## Abstract

Type 2 diabetes and hypertension frequently coexist, necessitating multi-target interventions. This study used a virtual-to-experimental pipeline to identify dual dipeptidyl peptidase-IV (DPP-IV)/angiotensin-converting enzyme (ACE) inhibitory peptides from Tartary buckwheat (*Fagopyrum tataricum*), identifying VPIY and IPLIGF. VPIY showed IC_50_ (half-maximal inhibitory concentration) values of 191.61 ± 4.99 μM for DPP-IV and 140.85 ± 4.35 μM for ACE, whereas IPLIGF showed 291.43 ± 4.29 μM and 199.85 ± 5.59 μM for DPP-IV and ACE, respectively. Kinetic analyses using global nonlinear mixed-model fitting revealed that VPIY acted as a mixed-type inhibitor of DPP-IV and a non-competitive inhibitor of ACE, whereas IPLIGF was non-competitive against DPP-IV and competitive-like against ACE. Molecular docking confirmed VPIY as a peripheral, Zn^2+^-non-coordinating ACE inhibitor, whereas IPLIGF ligated the ACE Zn^2+^, supporting competitive inhibition. Notably, VPIY displayed ~5-fold enhancement over its enzymatic IC_50_ potency in Caco-2 (human colorectal adenocarcinoma) cells (IC_50_ = 38.03 ± 2.23 μM). Both peptides restored nitric oxide (NO) and suppressed endothelin-1 (ET-1) in norepinephrine-injured human umbilical vein endothelial cells (HUVECs), mitigating endothelial dysfunction. These first-reported dual-inhibitory peptides from Tartary buckwheat, particularly VPIY, exert potent in situ DPP-IV inhibitory activity on Caco-2 cells and vascular protection, supporting their development as food ingredients for metabolic syndrome.

## 1. Introduction

The global prevalence of type 2 diabetes mellitus (T2DM) and hypertension continues to rise, frequently converging within the metabolic syndrome phenotype to synergistically exacerbate cardiovascular complications and renal failure [1,2]. Although clinically effective, long-term administration of DPP-IV inhibitors (e.g., sitagliptin) and ACE inhibitors (e.g., captopril) is frequently accompanied by adverse effects—gastrointestinal disturbances for sitagliptin [3] and hyperkalemia for captopril, particularly in patients with renal impairment or diabetes [4]. This has fueled an escalating demand for safe, naturally derived bioactive compounds suitable for dietary intervention. Food-derived bioactive peptides, characterized by small size, high absorbability, multifunctional bioactivities, and favorable safety profiles, have emerged as prime candidates for managing metabolic disorders [5,6].

Tartary buckwheat (*Fagopyrum tataricum* (L.) Gaertn.), a traditional pseudo-cereal and ethnic food crop cultivated extensively in Southwest China, is recognized as a medicinal and edible homologous crop rich in high-quality protein, flavonoids, and D-chiro-inositol [7]. Recent studies have identified DPP-IV inhibitory peptides from Tartary buckwheat protein hydrolysates—e.g., LHIVGPDK (IC_50_ = 1.61 mM) [8] and the simulated-gastrointestinal-digestion-derived peptides LAGQS and LREIDDADK [9]—and have further evaluated their in situ inhibitory effects using Caco-2 monolayers. Concurrently, ACE inhibitory peptides have been isolated from Tartary buckwheat albumin, yielding 42 active peptides with potent inhibitory potential [10]. However, to the best of our knowledge, no study has yet reported DPP-IV/ACE dual-functional peptides derived from Tartary buckwheat, despite the reported mono-functional peptide libraries for both target enzymes.

The screening of dual-functional peptides is intrinsically challenging due to the divergent structural preferences of the two target enzymes: DPP-IV typically favors short peptides with Pro or hydrophobic residues at the N-terminus, whereas ACE preferentially binds peptides with aromatic or hydrophobic amino acids at the C-terminus [11,12]. Consequently, natural sequences satisfying both recognition motifs are rare. While virtual screening has accelerated the discovery of mono-functional peptides [13,14,15,16,17,18], its application to dual inhibitors has been explored in only a few systems. Representative advances include: bitter gourd seed protein, which yielded the dual-functional peptide APLVSW (ACE IC_50_ = 9.6 μM, DPP-IV IC_50_ = 145.4 μM) via orthogonal bioassay-guided fractionation [19]; sea cucumber viscera, which provided WEPFP, GFPGLP, FPGGPP, and LPPGPFP as ACE-inhibitory peptides with concurrent DPP-IV inhibitory activity [20]; Bambara groundnut hydrolysates, where in silico screening combined with molecular docking identified candidate dual inhibitors such as EPWWPK [21]; and quinoa protein hydrolysates, which exhibited multifunctional inhibition of DPP-IV, ACE, and α-glucosidase [22]. Furthermore, sericin-derived peptides have demonstrated stability of dual DPP-IV/ACE inhibitory activity during in vitro gastrointestinal digestion [23]. Nevertheless, three persistent limitations constrain the translational potential of current dual-functional peptide research: (i) most studies rely on labor-intensive hydrolysate fractionation with low hit rates [19,20]; (ii) the intrinsic divergence in DPP-IV and ACE substrate recognition makes natural dual-inhibitory sequences exceptionally rare [11,12]; and (iii) the majority of prior works lack cellular-level validation of inhibitory activity and vascular endothelial protection, leaving the in vivo relevance of identified peptides largely unexplored.

We hypothesized that a targeted virtual-to-experimental pipeline, anchored by a pre-validated library of DPP-IV inhibitory peptides from Tartary buckwheat, could efficiently overcome the low hit-rate bottleneck and enable the rational discovery of novel dual-functional peptides. In this study, we integrated in silico structural filtering, dual-target predictive modeling (StackDPPIV [24] and AHTpin [25]), physiochemical and safety profiling, and solid-phase synthesis to screen candidate peptides. Subsequently, we employed a comprehensive suite of biochemical and cellular assays—including enzymatic kinetics, molecular docking, DPP-IV inhibitory assay in Caco-2 cells, and HUVEC-based endothelial function assays—to systematically characterize the inhibitory mechanisms and biological potential of the identified peptides. This work aims to expand the functional peptide repertoire of Tartary buckwheat and provide a scientific basis for developing functional foods targeting metabolic syndrome.

## 2. Materials and Methods

### 2.1. Materials and Reagents

Synthetic peptides VPIY and IPLIGF (purity ≥ 95%) were custom-synthesized and purified from Sangon Biotech (Shanghai, China). Sitagliptin (DPP-IV positive control) and Captopril (ACE positive control) were obtained from Yuanye Bio-Technology (Shanghai, China). DPP-IV (≥10 U/mg), ACE (≥0.1 U/mg), Gly-Pro-*p*-nitroanilide (Gly-Pro-*p*NA), and N-[3-(2-furyl)acryloyl]-L-phenylalanylglycylglycine (FAPGG) were purchased from MedChemExpress (Monmouth Junction, NJ, USA). Dulbecco’s Modified Eagle Medium (DMEM), fetal bovine serum (FBS), and cell culture supplements were from Gibco (Thermo Fisher Scientific, Waltham, MA, USA). Nitric oxide (NO) assay kits and human endothelin-1 (ET-1) ELISA kits were sourced from Nanjing Jiancheng Bioengineering Institute (Nanjing, China). Caco-2 cells (Cat. No. GCL-0050) and HUVECs (PUMC-HUVEC-T1, SV40T-immortalized human umbilical vein endothelial cells, Cat. No. CL-0675) were obtained from Procell Life Science & Technology (Wuhan, China).

### 2.2. Bioinformatic Analysis

The DPP-IV inhibitory potential of filtered peptides was re-evaluated using the StackDPPIV online platform (http://pmlabstack.pythonanywhere.com/StackDPPIV, accessed on 12 March 2026) [24], retaining peptides with scores ≥0.5. ACE inhibitory potential was predicted using the AHTpin server (https://webs.iiitd.edu.in/raghava/ahtpin/, accessed on 12 March 2026) [25], retaining peptides with SVM scores > 0. The Grand Average of Hydropathy (GRAVY) was calculated using the GRAVY Calculator (Kyte-Doolittle scale). Isoelectric points (pI) were predicted using Isoelectric Point Calculator 2.0 (http://www.ipc2-isoelectric-point.org, accessed on 12 March 2026). Peptides with GRAVY > 0 and pI between 4.0 and 8.0 were retained. Toxicity and allergenicity were assessed using ToxinPred 1.0 (https://webs.iiitd.edu.in/raghava/toxinpred/, accessed on 12 March 2026) [26] and AllerCatPro 2.0 (https://allercatpro.bii.a-star.edu.sg/, accessed on 12 March 2026) [27], respectively; toxic or allergenic peptides were excluded.

### 2.3. Solid-Phase Peptide Synthesis

Candidate peptides were synthesized via standard Fmoc solid-phase peptide synthesis (SPPS) by Sangon Biotech (Shanghai, China). Wang resin was used as the solid support. Deprotection was achieved using 20% piperidine in DMF. Amino acid coupling was facilitated by HBTU/HOBt/DIEA activation. Following chain elongation, peptides were cleaved from the resin using trifluoroacetic acid (TFA), precipitated with cold diethyl ether, and purified via reverse-phase high-performance chromatography (RP-HPLC) on a C18 column using an acetonitrile/water gradient. Desalting was performed either during RP-HPLC or via subsequent C18 solid-phase extraction to remove residual salts and TFA. Purified fractions were verified by electrospray ionization mass spectrometry (ESI-MS) to confirm the expected molecular weight, and purity was assessed by analytical HPLC, yielding peptides with ≥95% purity. The purified peptides were lyophilized and stored at −20 °C until use.

### 2.4. In Vitro Enzymatic Activity Assays

#### 2.4.1. Dual-Activity Pre-Screening

Synthesized peptides (1 mg/mL) were preliminarily screened for simultaneous inhibition of DPP-IV and ACE. Peptides exhibiting >30% inhibition against both enzymes proceeded to detailed characterization.

#### 2.4.2. DPP-IV Inhibition Assay

DPP-IV inhibitory activity was evaluated using a modified version of a previously reported method [28]. Reactions (100 μL total volume) contained 25 μL peptide sample (in 0.1 M Tris-HCl, pH 8.0), 25 μL 1.6 mM Gly-Pro-*p*NA. After pre-incubation at 37 °C for 10 min, the reaction was initiated by adding 50 μL DPP-IV (10 U/L) in the same buffer and incubated at 37 °C for 60 min. It was terminated by adding 100 μL of 1 M sodium acetate buffer (pH 4.0). The released p-nitroaniline was measured at 405 nm. Sitagliptin was used as the positive control. The inhibition rate (%) was calculated as:

(1) $$\mathrm{DPP-IV}\;\mathrm{inhibition}\;\mathrm{rate}\;(\%)\;=\;(1-\frac{A_{1}-A_{2}}{A_{3}-A_{4}})\;\times \;100\%$$

where *A*_1_: sample + substrate + enzyme; *A*_2_: sample + substrate (no enzyme); *A*_3_: substrate + enzyme (no sample, 100% activity); *A*_4_: substrate only (no sample, no enzyme). All groups were adjusted to the same final volume with 0.1 M Tris-HCl buffer (pH 8.0). All measurements were performed in triplicate.

#### 2.4.3. ACE Inhibition Assay

ACE inhibitory activity was determined using a modified protocol of a previously reported method [29]. Each 100 μL reaction contained 40 μL peptide sample in 80 mM HEPES, pH 8.3, 0.3 M NaCl, 50 μL 1 mM N-[3-(2-furyl) acryloyl]-L-phenylalanyl glycylglycine (FAPGG) substrate, and 10 μL ACE (0.1 U/mL) in the same buffer. After pre-incubation at 37 °C for 5 min, reactions were initiated by adding ACE and the decrease in absorbance at 340 nm was recorded for 30 min. Captopril served as the positive control. The inhibition rate (%) was calculated as:

(2) $$\mathrm{ACE}\;\mathrm{inhibition}\;\mathrm{rate}\;(\%)\;=\;\frac{\Delta A_{1}-\Delta A_{2}}{\Delta A_{1}}\times 100\%$$

where $\Delta A_{1}$ and $\Delta A_{2}$ are the decreases in absorbance at 340 nm over 30 min for the blank control (no inhibitor) and the sample group, respectively. All measurements were performed in triplicate.

#### 2.4.4. IC_50_ Determination

IC_50_ values were determined from concentration–response curves generated using serial dilutions of peptides. The tested peptide concentration ranges were 0.0078125–1.0000 mg/mL for the DPP-IV inhibition assay and 0.06250–2.0000 mg/mL for the ACE inhibition assay. Peptide concentrations were converted to μM based on their molecular weights (VPIY: 490.6 Da; IPLIGF: 657.8 Da) prior to analysis. The logit model was applied for dose–response fitting:

(3) $$\mathrm{logit}(p)\;=\;\ln(\frac{p}{1-p})\;=\;a\cdot log10C\;+\;b\;$$

where *p* is the proportion of inhibition (inhibition rate divided by 100, ranging from 0 to 1), *C* is peptide concentration in μM, *a* is the slope (regression coefficient) of the linear regression, and b is the y-intercept. The IC_50_ was calculated by solving the model equation when *p* = 0.5, which yields logit (0.5) = 0 and thus IC_50_ =$\;10^{-b/a}$. Each curve was generated from three independent experiments performed in triplicate. Goodness of fit was assessed using Nagelkerke pseudo-R^2^, with values ≥0.95 considered indicative of excellent model fit. The 95% confidence intervals (95% CIs) for IC_50_ were calculated by the profile likelihood method. All analyses were performed using GraphPad Prism 10 (GraphPad Software, San Diego, CA, USA). IC_50_ values are reported as mean ± SD.

#### 2.4.5. Inhibition Kinetics

The initial reaction velocities were determined at five varying substrate concentrations: Gly-Pro-*p*NA, (0.25–4.0 mM) for DPP-IV and FAPGG (0.25–3.0 mM) for ACE. Reactions were conducted in the absence (control) and presence of synthetic peptides at final concentrations corresponding to 0, 1/2 IC_50_, and IC_50_. The relationship between the initial reaction velocity (*V*) and substrate concentration ([*S*]) is described by the Michaelis–Menten equation:

(4) $$V\;=\;\frac{V_{\max}[S]}{K_{m}+[S]}$$

where $V_{\max}$ represents the maximum reaction velocity and $K_{m}$ is the Michaelis constant. To elucidate the inhibition type and calculate the kinetic parameters, the Lineweaver–Burk double-reciprocal method was applied. By taking the reciprocal of the Michaelis–Menten equation, the following linear relationship is obtained:

(5) $$\frac{1}{V}\;=\;\frac{K_{m}}{V_{\max}}\cdot (1+\frac{\left[I\right]}{K_{i}})\cdot \frac{1}{\left[S\right]}+\frac{1}{V_{\max}}\cdot (1+\frac{\left[I\right]}{\alpha K_{i}})$$

This equation conforms to a simple linear regression model (y = ax + b), where plotting $\frac{1}{V}$ (*y*-axis) against $\frac{1}{\left[S\right]}$ (*x*-axis) yields a straight line. The slope is $\frac{K_{m}}{V_{\max}}\cdot (1+\frac{\left[I\right]}{K_{i}})$, the x-intercept is $\frac{1}{V_{\max}}+\frac{\left[I\right]}{{V_{\max}K}_{i}}$, and the y-intercept is $\frac{1}{V_{\max}}\cdot (1+\frac{\left[I\right]}{\alpha K_{i}})$. *K_i_* denotes the inhibition constant of the inhibitor binding to free enzyme, and α describes the relative affinity of the inhibitor toward the enzyme–substrate complex. Based on the intersection patterns of the regression lines obtained at different peptide concentrations, the inhibition mechanisms were classified as competitive, non-competitive, or mixed-type. All kinetic parameters were derived from the global nonlinear regression analysis using GraphPad Prism 10 (GraphPad Software, San Diego, CA, USA).

### 2.5. Molecular Docking

Crystal structures of DPP-IV (PDB ID: 3W2T) [30] and ACE (PDB ID: 1O8A) [31] were retrieved from the RCSB Protein Data Bank. Water molecules and non-essential heteroatoms were removed using PyMOL 3.0.0 (Schrödinger, New York, NY, USA), while the catalytic Zn^2+^ ion was retained in the ACE structure to preserve the tetrahedral coordination geometry of the active site. Receptors were prepared using AutoDock Vina 1.5.7 by adding polar hydrogens and assigning Gasteiger charges, treated as rigid bodies, and saved in PDBQT format. Three-dimensional structures of VPIY and IPLIGF were constructed using ChemDraw 23.1.1 and Chem3D 23.1.1 (PerkinElmer, Waltham, MA, USA), with protonation states assigned at pH 7.0 (neutral C-terminal COOH, neutral N-terminal NH_2_; the Tyr residue in VPIY was modeled with a neutral phenolic hydroxyl group), followed by conversion to PDBQT format.

Molecular docking simulations were performed using AutoDock Vina 1.5.7. For DPP-IV, the search box was centered at (43.2, 59.2, 29.8) Å within the substrate-binding cavity; for ACE, the box was centered at (43.8, 38.3, 46.7) Å, directly encompassing the Zn^2+^-dependent active site, with dimensions of 40 × 40 × 40 Å^3^ for both enzymes. Exhaustiveness was set to 8, and the top 9 poses were generated for each complex. Hydrogen bonds were identified using a distance cutoff of ≤3.5 Å between donor and acceptor atoms (PyMOL polar contact analysis), with default angle cutoffs. Final poses were selected by sequential, kinetics-independent criteria: (i) energetic equivalence—poses within 2.0 kcal/mol of the top-scoring pose were shortlisted; (ii) pocket localization—poses had to lie within the defined box and within the catalytic/substrate cavity or immediate periphery, defined by distances to established active-site residues (DPP-IV: Arg125, Ser630, Tyr622, His740, Asn710; ACE: Zn^2+^, His383, His387, Glu411, Ala356, Gln281, Lys511, Arg522; Zn-ligand distance 1.9–2.6 Å, key H-bonds ≤3.5 Å); (iii) interaction priority—poses engaging the substrate-binding core, catalytic residues, or ACE Zn^2+^ coordination were preferred for canonical active-site inhibitors (e.g., captopril, enalaprilat [32]). In addition, poses contacting peripheral/allosteric residues without occupying the substrate core were accepted when structurally plausible.

Based on these criteria, for VPIY–ACE, the top-ranked pose (−8.5 kcal/mol) was selected, as it bound peripherally without direct Zn^2+^ coordination, engaging peripheral residues (list them) rather than the catalytic core. For IPLIGF–ACE, among energetically comparable poses (top pose: −8.3 kcal/mol), the third-ranked pose (−8.1 kcal/mol) was selected because it uniquely satisfied the Zn^2+^-coordination criterion (C-terminal carboxylate oxygen at 2.2 Å) and exhibited canonical ACE-inhibitor contacts. Experimental inhibition kinetics were not used as a selection criterion; the agreement between the selected pose and the observed competitive mechanism served as an independent validation. Visualization of docked complexes was performed using PyMOL 3.0.0. 

### 2.6. DPP-IV Inhibitory Assay in Caco-2 Cells

DPP-IV activity in Caco-2 cells was assessed using an adapted protocol from a previously reported method [33]. Caco-2 cells were seeded in 96-well plates at 2 × 10^4^ cells/well and cultured for 24 h. The cell layers were washed twice with sterile PBS. Cells were then incubated with 25 μL of peptide solutions (0–2 mg/mL) in 125 μL PBS at 37 °C for 10 min. Subsequently, 50 μL of 1 mM Gly-Pro-*p*NA was added, and incubation continued for 60 min. Absorbance was measured at 405 nm. Background (cells + peptide, no substrate), negative control (cells, no peptide), and blank (no cells, no peptide) groups were included. DPP-IV inhibition rates and IC_50_ values were calculated as described in Section 2.4.2. All experiments were performed in triplicate.

### 2.7. HUVEC Endothelial Protection Assay

HUVECs (3–5 passages) were seeded in 6-well plates at 2 × 10^5^ cells/well and cultured in human umbilical vein endothelial (primary cell immortalization) cell complete medium (Cat. No. CM-H082Y; Procell Life Science & Technology) at 37 °C under 5% CO_2_ until reaching 50–60% confluence. To induce endothelial dysfunction, cells were pretreated with 0.5 μM norepinephrine (NE) for 12 h, then co-incubated with VPIY or IPLIGF (100 or 200 μM) for 24 h. Captopril (1 μM) served as the positive control; untreated cells and NE-only (0.5 μM) cells functioned as blank and model controls, respectively. A combination group received both 200 μM peptide and 0.5 μM NE for 24 h to evaluate sustained efficacy. Supernatants were collected, centrifuged at 1000× *g* for 10 min at 4 °C, and stored at −80 °C. NO levels were measured by Griess assay and ET-1 levels by ELISA (both kits from Nanjing Jiancheng Bioengineering Institute, China) according to the manufacturers’ protocols. All experiments were performed in triplicate.

### 2.8. Cytotoxicity Assessment

Cell viability was evaluated using the CCK-8 assay. Caco-2 cells and HUVECs were seeded in 96-well plates at densities of 2 × 10^4^ and 8000 cells/well, respectively, and cultured for 24 h to allow adherence. Cells were then exposed to peptide solutions (Caco-2: 0–2 mg/mL; HUVECs: 100–400 μM, dissolved in sterile PBS) for 24 h. Following treatment, 10 μL of CCK-8 reagent was added to each well, and plates were incubated at 37 °C for 2 h. Absorbance was measured at 450 nm using a microplate reader. Cell viability (%) was calculated using the following formula:

(6) $$\mathrm{Cell}\;\mathrm{viability}\;(\%)\;=\;(\frac{A_{1}-A_{2}}{A_{3}-A_{2}})\;\times \;100\%$$

where $A_{1}$ is the absorbance of the experimental group (cells + peptide), $A_{2}$ is the absorbance of the blank control (medium + CCK-8, no cells), and $A_{3}$ is the absorbance of the vehicle control (cells + solvent, no peptide). All measurements were performed in triplicate.

### 2.9. Statistical Analysis

All experiments were performed in triplicate, representing three independent biological replicates (*n* = 3), with three technical replicates each. Data are presented as mean ± standard deviation (SD). For multiple-group comparisons, statistical significance was assessed by one-way analysis of variance (ANOVA) followed by Tukey’s post hoc test using OriginPro 2021 (OriginLab Corp., Northampton, MA, USA). A *p*-value < 0.05 was considered statistically significant. Specific inter-group comparisons (e.g., versus the Control group or the NE-treated group) and their corresponding significance annotations are detailed in the figure legends.

## 3. Results

### 3.1. Virtual Screening of Bifunctional Peptides

**Figure 1:**
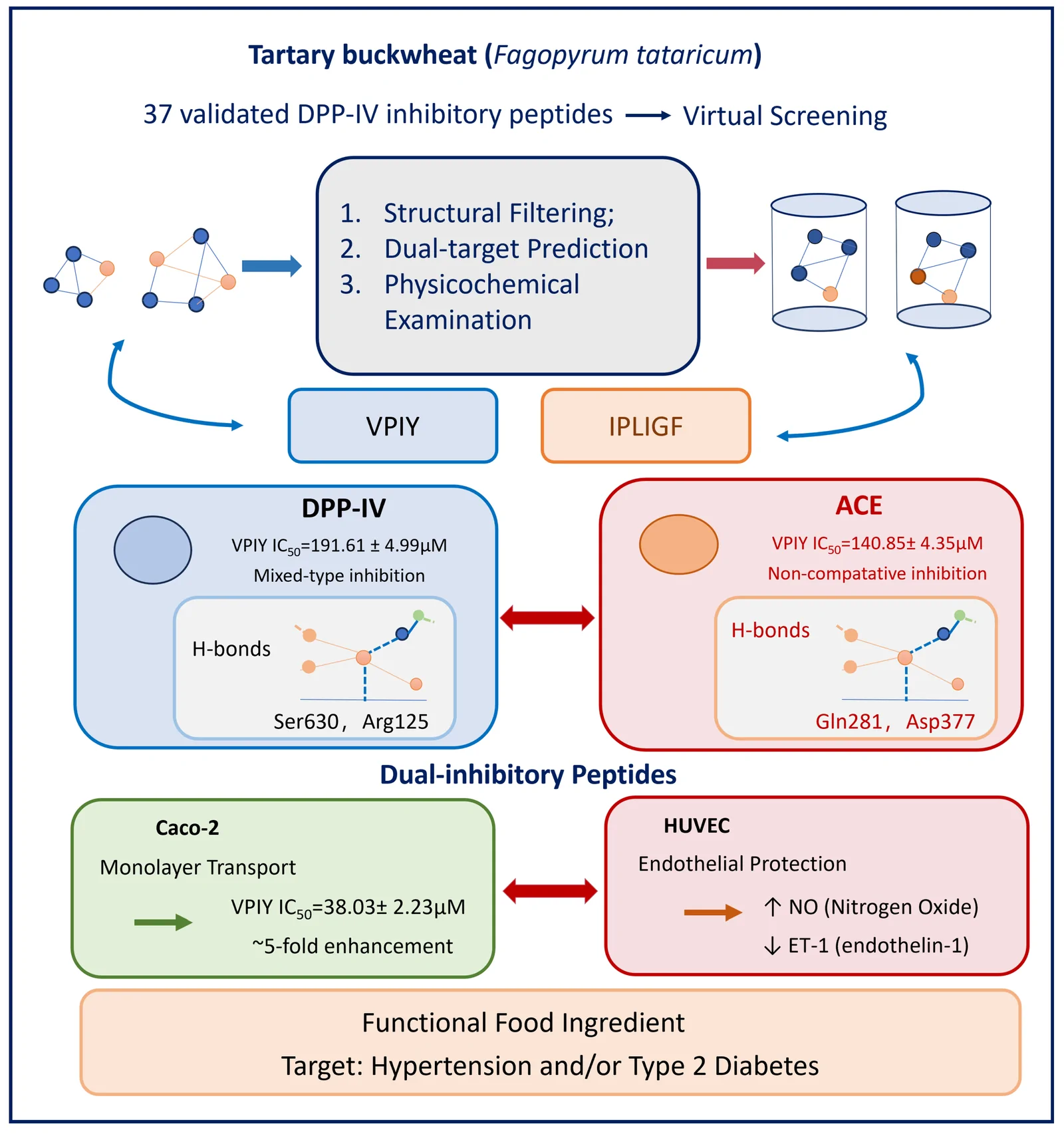
The discovery workflow of novel DPP-IV/ACE dual-inhibitory peptides from Tartary buckwheat (*Fagopyrum tataricum*).

To efficiently identify novel peptides with dual inhibitory activities against DPP-IV and ACE, a sequential in silico filtering pipeline was implemented, integrating structural feature analysis, dual-target predictive modeling, and physiochemical and safety profiling (Figure 1).

#### 3.1.1. Structural Features and Dual-Target Activity Prediction

Initially, 37 previously validated DPP-IV inhibitory peptides from Tartary buckwheat were subjected to filtering based on established ACE-inhibitory motifs, including chain lengths of 3–8 residues, specific N-terminal residues (Val, Ile, Leu, Phe, or Trp), and ≥50% hydrophobic amino acid composition. This step retained 16 candidate peptides. Subsequent dual-target prediction utilizing StackDPPIV, BIOPEP, and AHTpin servers revealed that StackDPPIV scores ranged from 0.66 to 1.00, while BIOPEP-derived DPP-IV scores (>0.50) corroborated prior experimental findings (Table S1). For ACE inhibition, BIOPEP scores ranged from 0.20 to 0.80, whereas AHTpin SVM scores varied considerably. Notably, VPIY (1.91), Seq12 (1.15), and Seq11 (1.09) exhibited high predicted ACE inhibitory potential (by AHTpin). Conversely, Seq13 (−0.54) and Seq14 (−0.05) returned negative SVM scores, indicating a low probability of ACE inhibition; these peptides were therefore excluded. No score was predicted for Seq2 by AHTpin; nevertheless, it was retained for further activity examination as a potential negative control. Ultimately, 14 peptides—13 with positive dual-target predictions and Seq2—advanced to subsequent evaluation.

#### 3.1.2. Physicochemical and Safety Profiling

The 14 candidate peptides were subjected to comprehensive physiochemical and safety assessments (Table S1). The Grand Average of Hydropathy (GRAVY) index, a measure of peptide hydrophobicity, ranged from 0.120 to 2.650. With the exception of VPVEK (0.120), all peptides exhibited GRAVY values ≥ 1.400, suggesting moderate to high hydrophobicity conducive to intestinal absorption and interaction with the hydrophobic pockets of target enzymes. The predicted isoelectric points (pI) ranged from 4.04 to 6.22, falling within the optimal physiological range (4.0–8.0), which ensures favorable solubility and charge balance in vivo. Crucially, in silico toxicological screening using ToxinPred 1.0 and AllerCatPro 2.0 predicted all 14 candidates to be non-toxic and non-allergenic, meeting the stringent safety requirements for food-derived bioactive peptides. Based on the integrated virtual screening workflow, these 14 peptides were ultimately selected for solid-phase synthesis and subsequent experimental validation.

### 3.2. Screening of Bifunctional Peptides

In vitro pre-screening at 1 mg/mL identified only two peptides—VPIY (Val-Pro-Ile-Tyr) and IPLIGF (Ile-Pro-Leu-Ile-Gly-Phe)—that concurrently inhibited DPP-IV and ACE by >30% (Figure S1). This low hit rate (5.4%, 2/37) underscores the challenge of identifying dual inhibitors due to the divergent substrate specificities of the two enzymes [11,12]. Nevertheless, this targeted screening approach proved substantially more efficient than random screening (<1% hit rate). These two peptides represent the first reported DPP-IV/ACE dual-inhibitory peptides originating from Tartary buckwheat.

### 3.3. Enzymatic IC_50_ Determination

**Figure 2:**
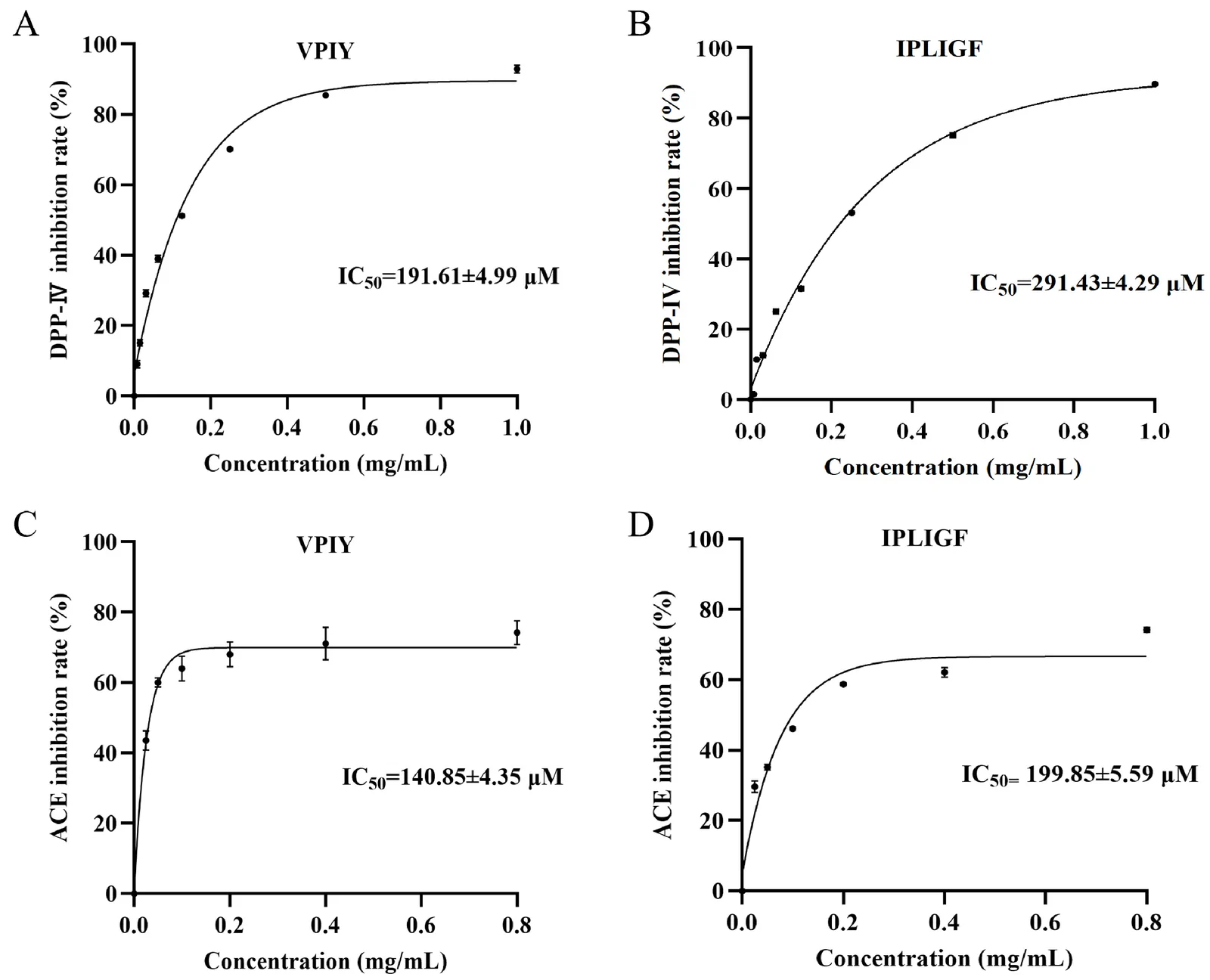
In vitro inhibitory activity of VPIY and IPLIGF against DPP-IV and ACE enzymes. DPP-IV inhibition rate curves for VPIY (**A**) and IPLIGF (**B**). ACE inhibition rate curves for VPIY (**C**) and IPLIGF (**D**).

In vitro DPP-IV and ACE enzymatic inhibitory assays were conducted to evaluate the antihypertensive and antidiabetic potential of VPIY and IPLIGF. As illustrated in Figure 2, both peptides exhibited dose-dependent inhibition against the target enzymes, with VPIY displaying relatively potent inhibitory activity compared to IPLIGF. The dose–response data were fitted to a logit model, which yielded Nagelkerke pseudo-R^2^ values ≥ 0.95 for all four peptide–enzyme combinations, indicating excellent model fit. Specifically, VPIY achieved half-maximal inhibition (IC_50_) values of 191.61 ± 4.99 µM (95% CI: 154.9–230.3 µM) against DPP-IV (Figure 2A) and 140.85 ± 4.35 µM (95% CI: 25.3–279.4 µM) against ACE (Figure 2C). In contrast, the longer hexapeptide IPLIGF exhibited lower inhibitory activities, with IC_50_ values of 291.43 ± 4.29 µM (95% CI: 217.1–402.2 µM) for DPP-IV (Figure 2B) and 199.85 ± 5.59 µM (95% CI: 139.6–296.0 µM) for ACE (Figure 2D).

### 3.4. Enhanced DPP-IV Inhibition on Caco-2 Cells

To further confirm the inhibitory effects of VPIY and IPLIGF on DPP-IV, an in situ experiment was conducted using Caco-2 cells (Table 1). Logit fitting gave Nagelkerke pseudo-R^2^ ≥ 0.95, indicating excellent fit. VPIY exhibited a remarkable IC_50_ of 38.03 ± 2.23 μM (95% CI: 27.20–50.11 μM) against DPP-IV, representing a ~5-fold enhancement in inhibitory potency compared to the cell-free enzymatic assay (191.61 ± 4.99 μM). Similarly, IPLIGF demonstrated an IC_50_ of 115.06 ± 1.89 μM (95% CI: 90.31–157.2 μM), which was substantially lower than its corresponding enzymatic IC_50_ (291.43 ± 4.29 μM). We observed that the IC_50_ values determined using the Caco-2 cell-based assay were lower than those obtained from the cell-free in vitro enzymatic assay. The reason for this difference remains to be fully elucidated, but it may reflect differences in peptide behavior between purified enzyme assays and cell-surface enzyme environments. Factors such as peptide-membrane interactions, local enzyme concentration at the cell surface, or differential peptide stability could contribute, as previously suggested [33,34,35]. Regardless, these findings collectively indicated that VPIY and IPLIGF exert DPP-IV inhibitory activity at the surface of Caco-2 cells.

### 3.5. Endothelial Protective Effects in HUVECs

**Figure 3:**
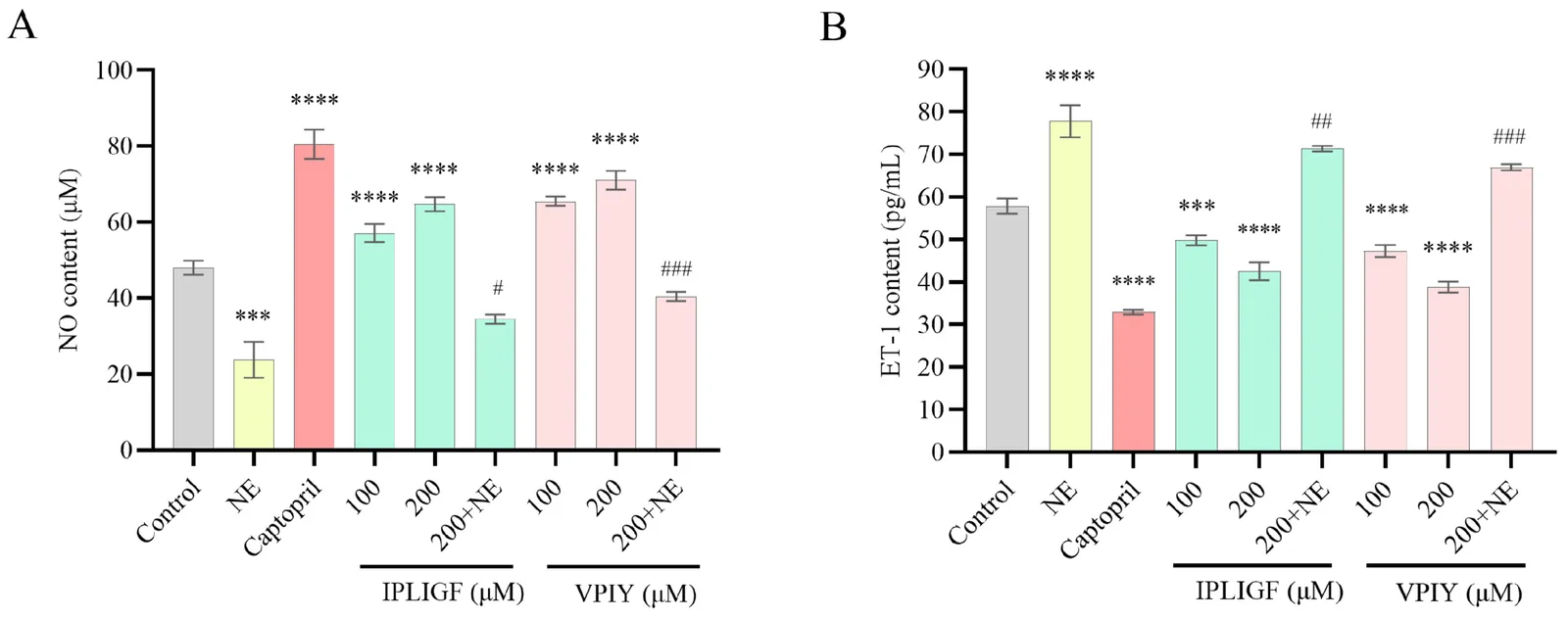
Effects of VPIY and IPLIGF on NO and ET-1 secretion in HUVECs. (**A**) NO content and (**B**) ET-1 content in HUVECs under different treatment conditions. Cells were pretreated with NE (0.5 μM) for 12 h to induce endothelial dysfunction, followed by co-treatment with VPIY or IPLIGF for another 24 h. Captopril (1 μM) was used as the positive control. Data are expressed as mean ± SD (*n* = 3). Statistical analysis: *** *p <* 0.001, **** *p* < 0.0001 vs. the Control group; ^#^ *p* < 0.05, ^##^ *p* < 0.01, ^###^ *p* < 0.001 vs. the NE-treated group.

Hypertension and diabetes inflict vascular damage partly through endothelial dysfunction, characterized by reduced NO bioavailability and ET-1 production [36,37]. Here, as shown in Figure 3, exposure to NE (0.5 μM) successfully induced endothelial dysfunction in HUVECs, as evidenced by a significant decrease in NO production (Figure 3A) and a concomitant increase in ET-1 production (Figure 3B) compared to the blank control (*p* < 0.05). In contrast, the positive control, captopril (1 μM), markedly reversed these alterations, confirming the robustness of the cellular model.

Notably, treatment with the bifunctional peptides VPIY and IPLIGF (100 and 200 μM) dose-dependently and significantly increased NO levels in HUVECs (Figure 3A), with VPIY exhibiting a more pronounced effect. This restoration of NO bioavailability suggests an enhanced endothelium-dependent vasodilation. Importantly, the protective effect persisted even under sustained adrenergic stimulation; co-incubation with 200 μM VPIY or IPLIGF alongside NE significantly elevated NO concentrations compared to the NE-only group (*p* < 0.05), indicating effective antagonism of NE-mediated NO inhibition and preservation of endothelial relaxation. Parallel trends were observed regarding ET-1 (Figure 3B), a potent vasoconstrictor. Both peptides dose-dependently reduced ET-1 concentrations (*p* < 0.05), with VPIY again showing superior efficacy. Under sustained NE stimulation, co-incubation with 200 μM peptides significantly attenuated aberrant ET-1 overproduction relative to the NE-only group (*p* < 0.05). Consistent with their respective enzymatic IC_50_ values, VPIY demonstrated more pronounced effects than IPLIGF in both NO restoration and ET-1 suppression under both basal and NE-stimulated conditions, underscoring its higher potency.

### 3.6. Cytocompatibility Assessment

**Figure 4:**
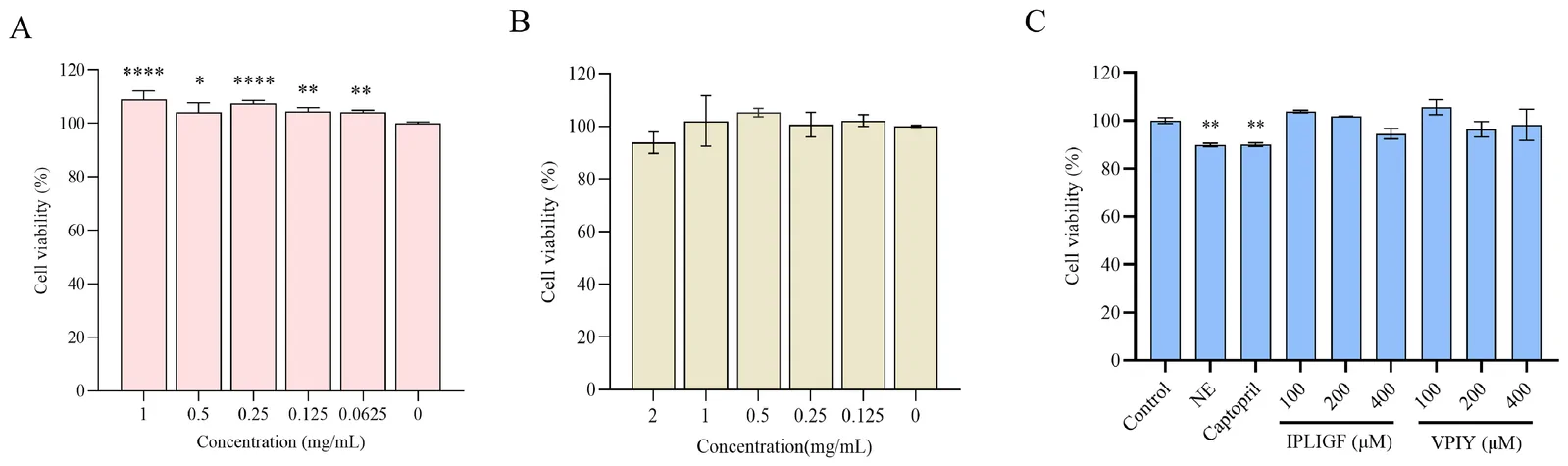
Cytocompatibility assessment of VPIY and IPLIGF in Caco-2 and HUVECs. Cell viability of Caco-2 cells treated with varying concentrations of VPIY (**A**) and IPLIGF (**B**). Comparative cytotoxicity assay in HUVECs (**C**). Data are expressed as mean ± SD (*n* = 3). * *p* < 0.05, ** *p* < 0.01, **** *p* < 0.0001 vs. the Control group. No marking indicating no significant difference vs. the Control group.

Prior to investigating the pharmacological effects, a comprehensive evaluation of the cytotoxic potential of VPIY and IPLIGF was conducted using the CCK-8 assay on Caco-2 and HUVECs, which represent the intestinal absorption and vascular endothelial models, respectively. As illustrated in Figure 4, treatment with VPIY and IPLIGF at concentrations ranging from 0.125 to 1 mg/mL respectively for 24 h did not significantly compromise the viability of Caco-2 cells compared to the untreated control (*p* > 0.05). Notably, the highest concentration tested (1 mg/mL) of VPIY maintained cell viability above 100%, suggesting that VPIY is well-tolerated by intestinal epithelial cells within this range. The cytocompatibility profile of the peptides in the vascular model is presented in Figure 4C. HUVECs exposed to NE (0.5 μM) and captopril (1 μM) displayed robust cell viability, compared to the blank control (Figure 4C). Notably, treatment with VPIY and IPLIGF across the concentration range of 100–400 μM elicited no significant cytotoxic effects, with cell viabilities consistently comparable to the Control group (*p* > 0.05). Importantly, even at the highest concentration of 400 μM—substantially exceeding the dosages required for the observed endothelial protective effects—both peptides retained excellent biocompatibility. Collectively, these findings confirm that VPIY and IPLIGF possess favorable cytocompatibility profiles in vascular endothelial cells, establishing a solid preclinical safety foundation for their development as potential nutraceuticals targeting metabolic syndrome. This non-toxic nature at effective concentrations establishes a solid preclinical safety foundation for their development as potential nutraceuticals targeting metabolic syndrome.

### 3.7. Enzymatic Inhibition Kinetics

**Figure 5:**
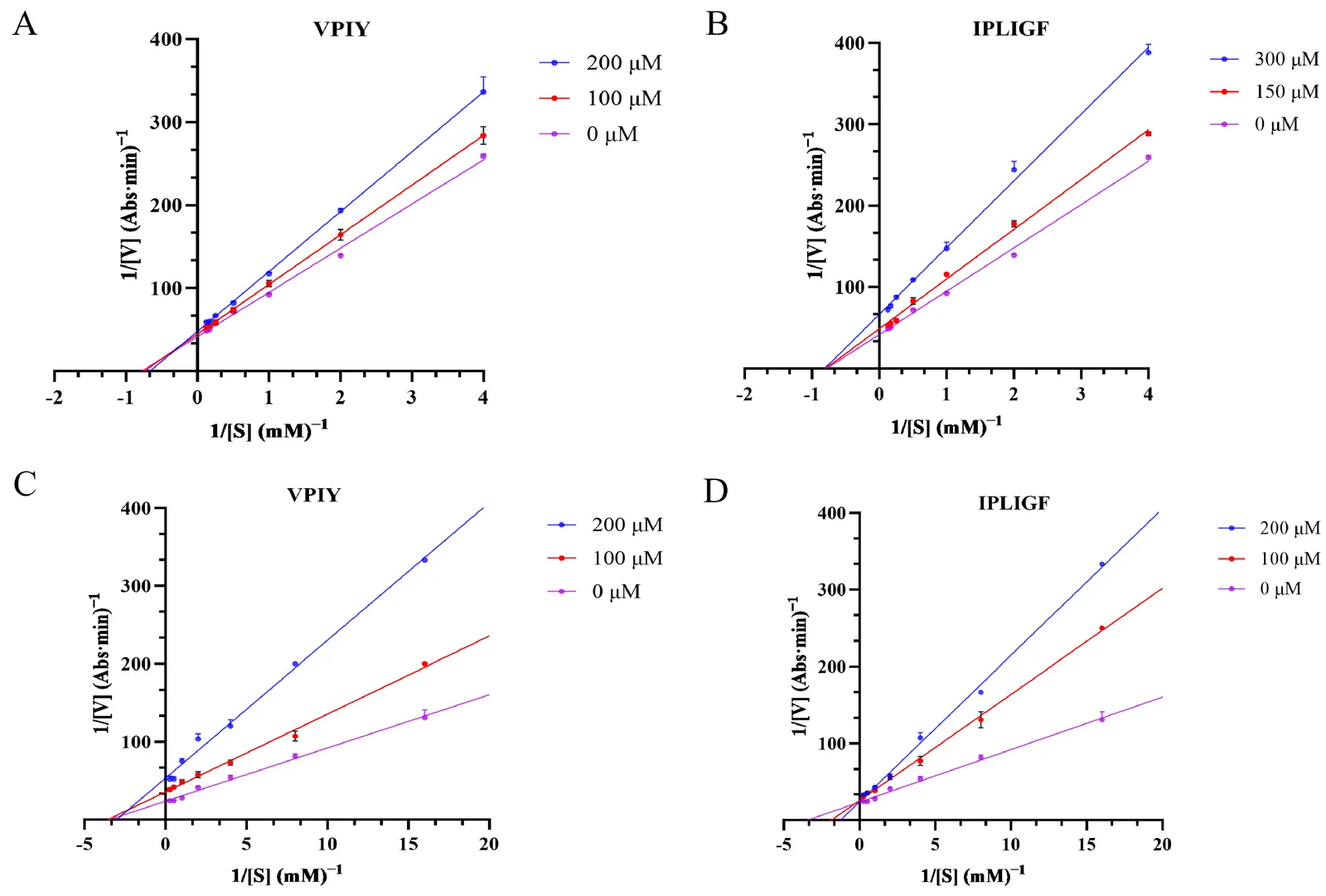
Lineweaver-Burk double-reciprocal plots illustrating the inhibition kinetics of VPIY and IPLIGF against DPP-IV (**A**,**B**) and ACE (**C**,**D**).

Kinetic analyses of VPIY and IPLIGF against DPP-IV and ACE were performed using global nonlinear regression with a mixed-inhibition model, which simultaneously estimated the shared kinetic parameters (*Ki* and *α*) from the substrate-velocity datasets (Table S2). This approach provided quantitative inhibition constants and allowed unambiguous assignment of inhibition mechanisms based on α values and their 95% confidence intervals (CIs). For qualitative comparison, Lineweaver-Burk (LB) double-reciprocal plots were also constructed (Figure 5), and their intersection patterns were fully consistent with the nonlinear fitting results, but inhibition types were definitively assigned by the global fit.

For VPIY against DPP-IV (Figure 5A), the global fit yielded α = 2.84 (95% CI: 1.72–5.12), which is significantly greater than 1 (CI excludes 1), indicating a mixed-type inhibition biased toward competitive character [38]. The *Ki* was estimated as 534.9 μM (95% CI: 443–661 μM). Consistently, the LB plots intersected in the second quadrant, suggesting that VPIY binds to both free enzyme and enzyme-substrate complex with different affinities, altering both apparent *K_m_* and *V_max_*. Against ACE, VPIY functioned as a non-competitive inhibitor (Figure 5C), with *α* = 1.10 (95% CI: 0.72–1.68; CI includes 1) and *Ki* = 144.3 μM (95% CI: 113–194 μM). The family of LB lines converged at a common x-intercept (−1/*K_m_*), signifying an unchanged *K_m_*, while the increased y-intercept (−1/*V_max_*) reflected a reduced *V_max_*. This kinetic signature indicates binding at a site distinct from the substrate-binding pocket, consistent with allosteric modulation of ACE catalysis [39].

For IPLIGF against DPP-IV (Figure 5B), the peptide acted as a non-competitive inhibitor, with α = 1.43 (95% CI: 0.86–2.42; CI includes 1) and *Ki* = 472.4 μM (95% CI: 367–642 μM). The LB plots showed convergence at the x-axis, indicative of allosteric modulation rather than direct active-site blockade [39]. Strikingly, IPLIGF exhibited competitive-like mixed inhibition against ACE (Figure 5D), with α = 6.84 (95% CI: 3.90–15.70; CI excludes 1) and *K_i_* = 131.0 μM (95% CI: 111–156 μM). Although the LB lines appeared to converge on the y-axis—suggesting unaltered *V_max_* and an increased apparent *K_m_*, typical of competitive inhibition—the global fit revealed an α value substantially greater than 1, confirming that IPLIGF binds predominantly to the free enzyme but also retains residual affinity for the ES complex. This kinetic behavior signifies direct competition with the substrate FAPGG for occupancy of the ACE active site, but with a mixed component likely arising from weak secondary interactions [38].

### 3.8. Molecular Docking Insights into Binding Modes

**Figure 6:**
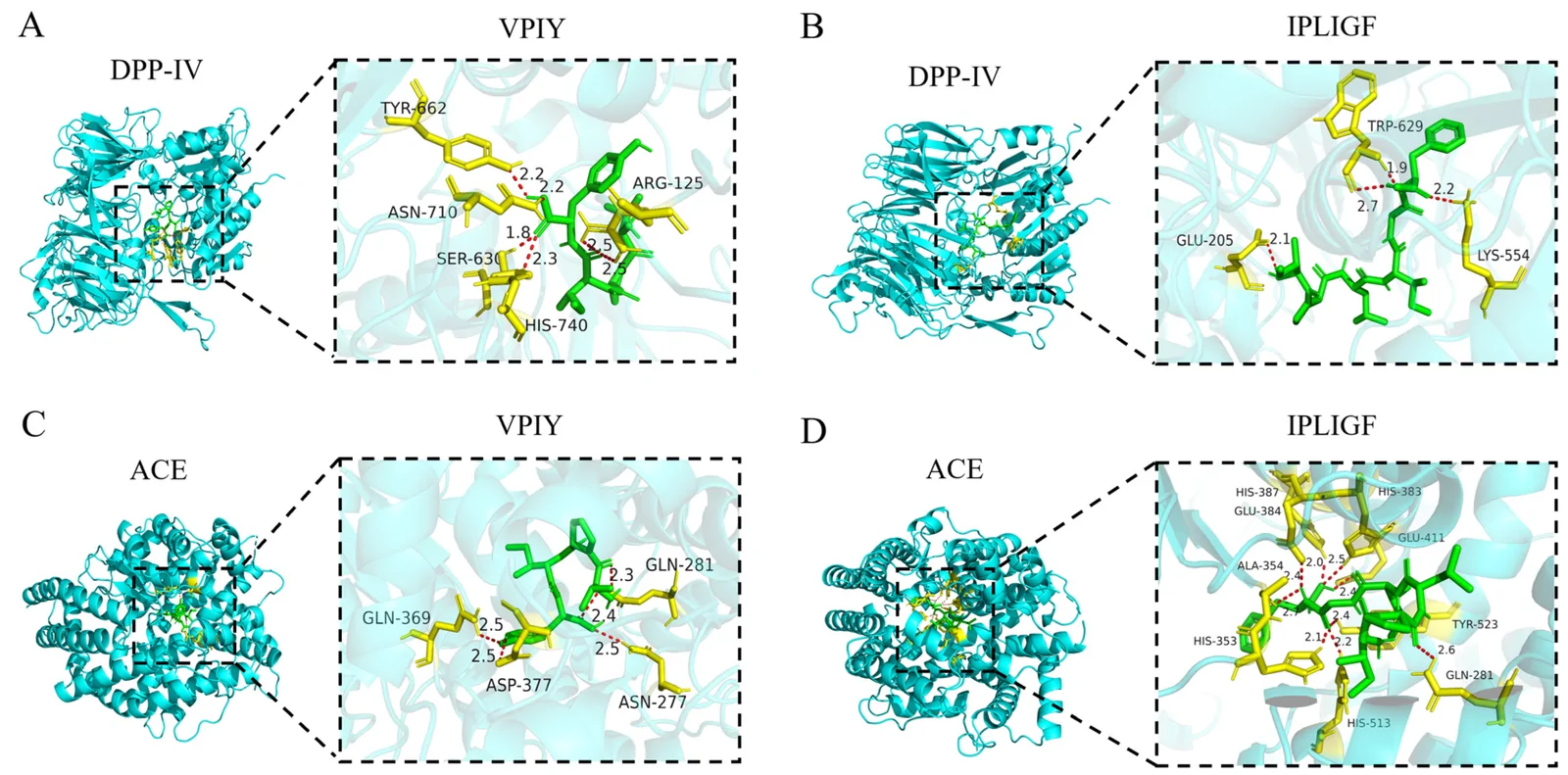
Docking conformation and interaction details of VPIY and IPLIGF with DPP-IV and ACE. Docking pose of VPIY (**A**) and IPLIGF (**B**) within the active site of DPP-IV; docking pose of VPIY (**C**) and IPLIGF (**D**) within the active site of ACE. The enzyme is depicted in cyan (surface and cartoon representation), the peptides are shown in green (stick representation), and the key interacting amino acid residues are shown in yellow (stick representation). Key interacting amino acid residues are labeled in black text.

Molecular docking simulations were performed to elucidate the molecular basis of the distinct inhibition profiles of VPIY and IPLIGF against DPP-IV and ACE (Figure 6, and Table 2).

DPP-IV binding modes. VPIY docked into the DPP-IV active cavity with a predicted binding affinity of −7.0 kcal/mol and formed six hydrogen bonds. The interaction network spanned both the S1′ pocket (Arg125, 2.5 Å) and the S1/S2 region (Ser630, 1.8 Å; Tyr622, 2.2 Å; His740, 2.3 Å; Asn710, 2.2 Å) [40,41]. Notably, simultaneous engagement of Arg125—a key determinant of P2-residue recognition—and Ser630, the catalytic nucleophile of the oxyanion hole, indicates that VPIY simultaneously contacts both the substrate-binding region and the catalytic apparatus [41]. In contrast, IPLIGF exhibited a stronger predicted binding affinity (−8.2 kcal/mol) but formed only four hydrogen bonds, exclusively with peripheral residues (Glu205, 2.1 Å; Lys554, 2.2 Å; Trp629, 2.7 Å and 1.9 Å) [41]. The absence of direct contact with the catalytic Ser630 distinguishes the IPLIGF-DPP-IV interaction pattern from that of VPIY.

ACE binding modes. VPIY bound to ACE with a predicted affinity of −8.5 kcal/mol and formed three hydrogen bonds exclusively with peripheral residues (Gln369, 2.4 Å; Lys511, 2.3 Å; Gln281, 2.4 Å) [42]. Critically, VPIY did not directly coordinate with the zinc ion (Zn^2+^), whose tetrahedral coordination sphere is maintained by His383, His387, and Glu411 [42,43]. This peripheral binding mode is mechanistically consistent with its non-competitive inhibition kinetics. IPLIGF, however, displayed a strong binding affinity (−8.3 kcal/mol) and an extensive binding network comprising five interactions, including direct contacts with the zinc-coordinating residues His383 (2.5 Å) and His387 (2.5 Å), as well as Ala356 (2.3 Å) and Arg522 (2.2 Å, 2.7 Å). IPLIGF directly ligates the active-site Zn^2+^ (2.2 Å) via its C-terminal carbonyl oxygen, occupying the fourth coordination site of the tetrahedral metal center. The direct occupation of the Zn^2+^-dependent catalytic core by IPLIGF stands in sharp contrast to the peripheral binding mode observed for VPIY, thereby supporting its competitive-like mixed inhibition profile.

## 4. Discussion

The present study identifies VPIY and IPLIGF as novel DPP-IV/ACE dual-inhibitory peptides—the first, to the best of our knowledge, to be reported from Tartary buckwheat, a traditional pseudo-cereal and ethnic food crop cultivated extensively in Southwest China, capable of concurrently inhibiting DPP-IV and ACE activity while restoring the NE-disrupted NO/ET-1 balance in HUVECs. Moreover, VPIY outperformed IPLIGF in cell-free ACE/DPP-IV kinetics, endothelial NO/ET-1 rescue in HUVECs, and Caco-2 cell-surface DPP-IV blockade, with molecular docking offering post hoc binding hypotheses consistent with these observations.

Mechanistic Convergence of ACE/DPP-IV Dual Inhibition on Endothelial Homeostasis. In Section 3.5, NE (0.5 μM) reduced NO and increased ET-1 in HUVECs, whereas captopril (1 μM) reversed both changes, validating the dysfunction model. In unchallenged HUVECs, VPIY and IPLIGF (100–200 μM) increased NO and decreased ET-1 concentration-dependently; in NE-insulted cells, 200 μM of either peptide partially restored NO bioavailability and attenuated ET-1 overproduction. Because both peptides were confirmed as direct ACE and DPP-IV inhibitors in cell-free kinetic assays, these phenotypes are interpretable within established ACE/DPP-IV endothelial responses. ACE inhibition may improve NO/ET-1 balance by lowering angiotensin II–dependent ROS and eNOS uncoupling [44] and by reducing bradykinin degradation to potentiate B2/eNOS signaling and NO release [45,46]; ACE inhibitors likewise decrease oxidative stress and tissue ET-1 in endothelial/vascular models [45,47]. DPP-IV inhibition may independently contribute by prolonging GLP-1/substance P [48,49]; in HUVECs, alogliptin increased eNOS Ser1177 and Akt Ser473 phosphorylation and NO via Src/Akt/eNOS [50], while other DPP-IV inhibitors attenuated inflammatory adhesion molecules through Akt/AMPK [51]. Thus, VPIY/IPLIGF-induced NO recovery and ET-1 reduction are consistent with dual ACE/DPP-IV engagement, but intracellular mediator levels and enzyme activity were not measured, so specific peptide-level mediation is not demonstrated. Human studies show context-dependent ACE–DPP-IV interactions rather than uniform additivity: sitagliptin modulated enalapril effects on blood pressure, renal blood flow and sympathetic tone [52], and combined inhibition can alter substance-P-mediated sympathetic responses [49]. Therefore, while the directional NO/ET-1 changes align with dual biochemical inhibition, alternative peptide-dependent mechanisms—direct antioxidant effects, adrenergic receptor interactions or non-enzymatic vasoactive actions—cannot be excluded. Future studies should quantify cellular ACE/DPP-IV activity, bradykinin, GLP-1, substance P, ROS and eNOS/Akt intermediates.

Structural and Kinetic Determinants of Differential Potency. VPIY consistently outperformed IPLIGF across enzymatic and cellular assays. In cell-free systems, VPIY exhibits lower IC_50_ values against DPP-IV (191.61 vs. 291.43 μM) and ACE (140.85 vs. 199.85 μM). In Caco-2 cells, VPIY exhibited a ~5-fold enhancement in cell-surface DPP-IV blockade (IC_50_ = 38.03 μM), surpassing IPLIGF’s ~2.5-fold improvement. In HUVECs, VPIY more effectively restored NO bioavailability and suppressed ET-1 overproduction under NE insult. This superiority stems from three interrelated determinants. First, structural and physicochemical properties. VPIY (tetrapeptide, 490.6 Da) is smaller than IPLIGF (hexapeptide, 657.8 Da), which may facilitate faster diffusion and easier active-site access in complex microenvironments [35]. Second, kinetic advantage. Global nonlinear regression (mixed-type model) yielded quantitative parameters explaining the potency differences. For DPP-IV, VPIY showed mixed-type inhibition biased toward competitive (*K_i_* = 534.9 μM, α = 2.84), while IPLIGF was non-competitive (*K_i_* = 472.4 μM, α = 1.43). For ACE, VPIY acted as a non-competitive inhibitor (*K_i_* = 144.3 μM, α = 1.10), whereas IPLIGF displayed competitive-like mixed inhibition (*K_i_* = 131.0 μM, α = 6.84). These results, in close agreement with Lineweaver-Burk intersection patterns (Figure 6), indicate that LB plots served only as qualitative cross-validation, with inhibition types definitively assigned by global fitting. Third, molecular docking provided structural hypotheses: IPLIGF’s C-terminal carboxylate coordinated the ACE Zn^2+^ (2.2 Å) with H-bonds to His383/His387, consistent with competitive blockade [41,53]; VPIY bound peripherally, avoiding Zn^2+^-coordinating residues, correlating with non-competitive behavior. This kinetic behavior parallels that of the yeast-derived hexapeptide TPTQQS, whose C-terminal residue disrupts the tetrahedral zinc coordination while its N-terminal residues engage the lid structure of ACE [39]. Analogous trends were observed for DPP-IV (VPIY engaged Arg125/Ser630). These in silico models represent interpretations of kinetic data rather than independent mechanistic evidence. Importantly, cellular efficacy in Caco-2 and HUVEC models likely reflects a combination of enzyme inhibition, peptide stability, and microenvironmental factors beyond binding affinity alone.

Bridging Computational Prediction and Experimental Reality. Notably, although IPLIGF formed a greater number of hydrogen bonds with ACE (five vs. three for VPIY) and directly ligated the catalytic Zn^2+^, its in silico binding affinity for the selected pose (−8.1 kcal/mol) was comparable to that of VPIY (−8.5 kcal/mol), and its in vitro IC_50_ values were higher than those of VPIY for both enzymes. This apparent discrepancy underscores a fundamental caveat in molecular docking studies: static docking scores reflect only the thermodynamic stability of the ligand-receptor complex under a rigid-receptor approximation and do not linearly correlate with dynamic enzymatic inhibition [54,55]. The actual inhibitory potency is governed by a multitude of factors—including induced-fit conformational dynamics, solvation effects, the presence of the natural substrate, cellular uptake efficiency, and peptidase stability [55]—that are not captured by a single-point docking score. Notably, this trend extended to the cellular level: in HUVEC-based endothelial protection assays, VPIY consistently outperformed IPLIGF in restoring NO bioavailability and suppressing ET-1 overproduction (Figure 4), mirroring the enzymatic IC_50_ hierarchy. This observation reinforces the necessity of integrating enzymatic kinetics, cellular assays, and computational docking to comprehensively evaluate peptide-based enzyme inhibitors, rather than relying solely on in silico affinity predictions.

Contextual Positioning Among Food-Derived Dual-Functional Peptides. Within the broader landscape of food-derived bioactive peptides, VPIY and IPLIGF exhibit moderate yet promising activities. Recent studies have identified DPP-IV inhibitory peptides from Tartary buckwheat protein hydrolysates (e.g., LHIVGPDK with IC_50_ = 1.61 mM; LAGQS and LREIDDADK with strong in vitro and in situ inhibition) [8,9], as well as 42 ACE inhibitory peptides from Tartary buckwheat albumin [10]. Relative to these, the dual DPP-IV/ACE inhibitory profile of VPIY (IC_50_ = 191.61 μM for DPP-IV and 140.85 ± 4.35 μM for ACE) suggests VPIY as a promising candidate within this specific class of pseudo-cereal-derived peptides. The true value of VPIY may lie in its cultural and dietary heritage as a traditional pseudo-cereal cultivated extensively in Southwest China and recognized as a medicinal and edible homologous crop—and its applicability in whole-food-based functional nutrition.

In summary, VPIY emerges as a particularly promising candidate, leveraging its compact tetrapeptide architecture to achieve dual ACE/DPP-IV inhibition and potent activity in cellular models. Collectively, these complementary observations support further investigation into VPIY as a nutraceutical candidate. Future studies should prioritize in vivo validation of its pharmacokinetics and anti-hypertensive/antidiabetic efficacy, as well as direct quantification of intracellular signaling mediators (e.g., PI3K/Akt/eNOS phosphorylation) to fully elucidate the mechanistic interplay.

## Figures and Tables

**Table 1:** DPP-IV inhibitory activity of oligopeptides in Caco-2 cells (IC_50_ values).

Peptides	MW (Da)	IC_50_ (μM)	IC_50_ (μg/mL)
VPIY	490.586	38.03 ± 2.23	18.65 ± 1.1
IPLIGF	658.83	115.06 ± 1.89	75.8 ± 1.25

**Table 2:** Molecular docking of oligopeptides with DPP-IV and ACEs.

Enzyme	Peptide	Binding Affinity (kcal/mol)	H-Bond Count	H-Bond Interactions
DPP-IV	VPIY	−7.0	6	ARG-125 (2.5 Å, 2.5 Å), HIS-740 (2.3 Å), ASN-710 (2.2 Å), SER-630 (1.8 Å), TYR-622 (2.2 Å)
	IPLIGF	−8.2	4	GLU-205 (2.1 Å), LYS-554 (2.2 Å), TRP-629 (2.7 Å, 1.9 Å)
ACE	VPIY	−8.5	3	GLN-369 (2.4 Å), LYS-511 (2.3 Å), GLN-281 (2.4 Å)
	IPLIGF	−8.3	5	HIS-383 (2.5 Å), ALA-356 (2.3 Å), HIS-387 (2.5 Å), ARG-522 (2.2 Å, 2.7 Å)

## Data Availability

The original contributions presented in this study are included in the article/Supplementary Materials. Further inquiries can be directed to the corresponding author.

## Supplementary Materials

The following supporting information can be downloaded at: https://www.mdpi.com/article/10.3390/foods15213912/s1, Figure S1: Initial screening of DPP-IV and ACE inhibitory activities of oligopeptides; Table S1: Integrated virtual screening profile of candidate peptides from tartary buckwheat; Table S2: Inhibition constants (Ki) and α values from global nonlinear mixed model fitting for VPIY and IPLIGF against DPP IV and ACE.

## References

[1] Cassataro G., Geraci G., Giusti M.A., Maida C., Maggio V., Rizzo M., Mattina A. (2026). The Cardiovascular Burden of Diabetes: Risk Factors, Clinical Phenotypes, and Personalized Cardiometabolic Management. J. Clin. Med..

[2] Tanaka A., Node K. (2022). Pathogenic connection between hypertension and type 2 diabetes: How do they mutually affect each other?. Hypertens. Res..

[3] Engel S.S., Round E., Golm G.T., Kaufman K.D., Goldstein B.J. (2013). Safety and Tolerability of Sitagliptin in Type 2 Diabetes: Pooled Analysis of 25 Clinical Studies. Diabetes Ther..

[4] Textor S.C., Bravo E.L., Fouad F.M., Tarazi R.C. (1982). Hyperkalemia in azotemic patients during angiotensin-converting enzyme inhibition and aldosterone reduction with captopril. Am. J. Med..

[5] Wang W.W., Yang W.J., Dai Y., Liu J.H., Chen Z.Y. (2023). Production of Food-Derived Bioactive Peptides with Potential Application in the Management of Diabetes and Obesity: A Review. J. Agric. Food Chem..

[6] Fernando I.P.S., Wu J.P. (2025). Food-derived bioactive peptides: The gateway to reach the full potential of food proteins for human health. Trends Food Sci. Technol..

[7] Zou L., Wu D.T., Ren G.X., Hu Y.C., Peng L.X., Zhao J.L., Garcia-Perez P., Carpena M., Prieto M.A., Cao H. (2023). Bioactive compounds, health benefits, and industrial applications of Tartary buckwheat (*Fagopyrum tataricum*). Crit. Rev. Food Sci. Nutr..

[8] Wu W.J., Wang L.B., Qiu J., Zhuang Z.Y., Qin F., Tan Q.L., Wang Y.L., Wu L.L. (2024). Exploring dipeptidyl peptidase-IV inhibitory peptides from Tartary Buckwheat protein: A study of hydrolysis, fractionation, and molecular interactions. J. Food Sci..

[9] Wu W.J., Qiu J., Wang L.B., Tan Q.L., Zhuang Z.Y., Wang Y.L., Wu L.L. (2025). Identification and characterization of two novel dipeptidyl peptidase-IV inhibitory peptides from the simulated gastrointestinal digestion of Tartary buckwheat proteins. Food Chem..

[10] Li Y.F., Yang N., Shi F., Ye F., Huang J.R. (2023). Isolation and identification of angiotensin-converting enzyme inhibitory peptides from Tartary buckwheat albumin. J. Sci. Food Agric..

[11] Nongonierma A.B., FitzGerald R.J. (2019). Features of dipeptidyl peptidase IV (DPP-IV) inhibitory peptides from dietary proteins. J. Food Biochem..

[12] Wu J., Aluko R.E., Nakai S. (2006). Structural requirements of Angiotensin I-converting enzyme inhibitory peptides: Quantitative structure-activity relationship study of di- and tripeptides. J. Agric. Food Chem..

[13] Liu Y.Y., Zhang Y., Liu M.H., Yu S.N., Tian Z.L., Li W.N., Han W.W. (2025). Virtual Hydrolysis-Based Screening of Wheat-Derived DPP-IV Inhibitory Peptides: A Mechanistic Analysis Integrating Cell Experiments and Molecular Dynamics Simulations. J. Agric. Food Chem..

[14] Gao W., Liu M., Wang Y. (2023). Isolation, virtual screening, action mechanisms, chelation with zinc ions, and stability of ACE-inhibitory peptides from ginkgo seed globulin. RSC Adv..

[15] Zhang M., Liu H.Y., Wang Y.N., Yu G.D., Liu M.Y., Lu Z.C., Mao F.J., Wu Z., Pan D.D., Tu M.L. (2026). Food-Derived Multi-Target Antihypertensive Peptides: Sources, Mechanisms and AI-Driven Strategies. Foods.

[16] Yu S.N., Liu Y.Y., Fu X.Q., Yan W.F., Gao W.W., Li W.N., Han W.W. (2026). Virtual screening of sweet peptides from milk protein and molecular dynamics simulations mechanism analysis. J. Dairy Sci..

[17] Agoni C., Fernández-Díaz R., Timmons P.B., Adelfio A., Gómez H., Shields D.C. (2025). Molecular Modelling in Bioactive Peptide Discovery and Characterisation. Biomolecules.

[18] Wang L., Wang L., Liu X., Lin X., Fei T., Zhang W. (2025). Seaweeds-derived proteins and peptides: Preparation, virtual screening, health-promoting effects, and industry applications. Crit. Rev. Food Sci. Nutr..

[19] Hung W.T., Sutopo C.C.Y., Wu M.L., Hsu J.L. (2023). Discovery and Characterization of a Dual-Function Peptide Derived from Bitter Gourd Seed Protein Using Two Orthogonal Bioassay-Guided Fractionations Coupled with In Silico Analysis. Pharmaceuticals.

[20] Liang Q.P., Zhou W., Peng S.Y., Liu Z.M., Liang Z.Y., Wang L.B., Mukhtar H., Mou H.J. (2025). Exploration of ACE inhibitory peptides from sea cucumber viscera with DPP-IV inhibitory activity: Virtual screening, characterization, and potential mechanism investigation. Int. J. Biol. Macromol..

[21] Saetang J., Haewphet T., Nilsuwan K., Benjakul S. (2025). ACE- and DPP-IV-Inhibitory Peptides from Bambara Groundnut Hydrolysate: Elucidation Using Computational Tools and Molecular Docking. Biology.

[22] Mudgil P., Kilari B.P., Kamal H., Olalere O.A., FitzGerald R.J., Gan C.Y., Maqsood S. (2020). Multifunctional bioactive peptides derived from quinoa protein hydrolysates: Inhibition of α-glucosidase, dipeptidyl peptidase-IV and angiotensin I converting enzymes. J. Cereal Sci..

[23] Sangsawad P., Katemala S., Pao D.N., Suwanangul S., Jeencham R., Sutheerawattananonda M. (2022). Integrated Evaluation of Dual-Functional DPP-IV and ACE Inhibitory Effects of Peptides Derived from Sericin Hydrolysis and Their Stabilities during In Vitro-Simulated Gastrointestinal and Plasmin Digestions. Foods.

[24] Charoenkwan P., Nantasenamat C., Hasan M.M., Moni M.A., Lio P., Manavalan B., Shoombuatong W. (2022). StackDPPIV: A novel computational approach for accurate prediction of dipeptidyl peptidase IV (DPP-IV) inhibitory peptides. Methods.

[25] Tahir R.A., Bashir A., Yousaf M.N., Ahmed A., Dali Y., Khan S., Sehgal S.A. (2020). In Silico identification of angiotensin-converting enzyme inhibitory peptides from MRJP1. PLoS ONE.

[26] Rathore A.S., Choudhury S., Arora A., Tijare P., Raghava G.P.S. (2024). ToxinPred 3.0: An improved method for predicting the toxicity of peptides. Comput. Biol. Med..

[27] Nguyen M.N., Krutz N.L., Limviphuvadh V., Lopata A.L., Gerberick G.F., Maurer-Stroh S. (2022). AllerCatPro 2.0: A web server for predicting protein allergenicity potential. Nucleic Acids Res..

[28] Zhang Y., Chen R., Ma H.Q., Chen S.W. (2015). Isolation and Identification of Dipeptidyl Peptidase IV-Inhibitory Peptides from Trypsin/Chymotrypsin-Treated Goat Milk Casein Hydrolysates by 2D-TLC and LC-MS/MS. J. Agric. Food Chem..

[29] Li G.H., Liu H., Shi Y.H., Le G.W. (2005). Direct spectrophotometric measurement of angiotensin I-converting enzyme inhibitory activity for screening bioactive peptides. J. Pharm. BioMed Anal..

[30] Nabeno M., Akahoshi F., Kishida H., Miyaguchi I., Tanaka Y., Ishii S., Kadowaki T. (2013). A comparative study of the binding modes of recently launched dipeptidyl peptidase IV inhibitors in the active site. Biochem. Biophys. Res. Commun..

[31] Natesh R., Schwager S.L., Sturrock E.D., Acharya K.R. (2003). Crystal structure of the human angiotensin-converting enzyme-lisinopril complex. Nature.

[32] Natesh R., Schwager S.L., Evans H.R., Sturrock E.D., Acharya K.R. (2004). Structural details on the binding of antihypertensive drugs captopril and enalaprilat to human testicular angiotensin I-converting enzyme. Biochemistry.

[33] Zan R., Wu Q., Chen Y., Wu G., Zhang H., Zhu L. (2023). Identification of Novel Dipeptidyl Peptidase-IV Inhibitory Peptides in Chickpea Protein Hydrolysates. J. Agric. Food Chem..

[34] Caron J., Domenger D., Dhulster P., Ravallec R., Cudennec B. (2017). Using Caco-2 cells as novel identification tool for food-derived DPP-IV inhibitors. Food Res. Int..

[35] Xu Q.B., Hong H., Wu J.P., Yan X.H. (2019). Bioavailability of bioactive peptides derived from food proteins across the intestinal epithelial membrane: A review. Trends Food Sci. Technol..

[36] Dubsky M., Veleba J., Sojakova D., Marhefkova N., Fejfarova V., Jude E.B. (2023). Endothelial Dysfunction in Diabetes Mellitus: New Insights. Int. J. Mol. Sci..

[37] Kostov K. (2021). The Causal Relationship between Endothelin-1 and Hypertension: Focusing on Endothelial Dysfunction, Arterial Stiffness, Vascular Remodeling, and Blood Pressure Regulation. Life.

[38] Chagas R.S., Marana S.R. (2026). A general model for analysis of linear and hyperbolic enzyme inhibition mechanisms. FEBS Open Bio.

[39] Ni H., Li L., Liu G., Hu S.Q. (2012). Inhibition Mechanism and Model of an Angiotensin I-Converting Enzyme (ACE)-Inhibitory Hexapeptide from Yeast (*Saccharomyces cerevisiae*). PLoS ONE.

[40] Engel M., Hoffmann T., Wagner L., Wermann M., Heiser U., Kiefersauer R., Huber R., Bode W., Demuth H.U., Brandstetter H. (2003). The crystal structure of dipeptidyl peptidase IV (CD26) reveals its functional regulation and enzymatic mechanism. Proc. Natl. Acad. Sci. USA.

[41] Liu W., Wang X.Y., Yang W.N., Li X.Y., Qi D.Y., Chen H.J., Liu H.N., Yu S., Pan Y.L., Liu Y. (2022). Identification, Screening, and Comprehensive Evaluation of Novel DPP-IV Inhibitory Peptides from the Tilapia Skin Gelatin Hydrolysate Produced Using Ginger Protease. Biomolecules.

[42] Wang R.D., Lu X., Sun Q., Gao J.H., Ma L., Huang J.N. (2020). Novel ACE Inhibitory Peptides Derived from Simulated Gastrointestinal Digestion in Vitro of Sesame (*Sesamum indicum* L.) Protein and Molecular Docking Study. Int. J. Mol. Sci..

[43] He R., Aluko R.E., Ju X.R. (2014). Evaluating Molecular Mechanism of Hypotensive Peptides Interactions with Renin and Angiotensin Converting Enzyme. PLoS ONE.

[44] Oak J.H., Cai H. (2007). Attenuation of angiotensin II signaling recouples eNOS and inhibits nonendothelial NOX activity in diabetic mice. Diabetes.

[45] Desideri G., Grassi D., Croce G., Bocale R., Tiberti S., Evangelista S., Necozione S., Di Orio F., Ferri C. (2008). Different effects of angiotensin converting enzyme inhibitors on endothelin-1 and nitric oxide balance in human vascular endothelial cells: Evidence of an oxidant-sensitive pathway. Mediat. Inflamm..

[46] Erdös E.G., Tan F., Skidgel R.A. (2010). Angiotensin I-converting enzyme inhibitors are allosteric enhancers of kinin B1 and B2 receptor function. Hypertension.

[47] Lob H., Rosenkranz A.C., Breitenbach T., Berkels R., Drummond G., Roesen R. (2006). Antioxidant and nitric oxide-sparing actions of dihydropyridines and ACE inhibitors differ in human endothelial cells. Pharmacology.

[48] Liu L., Liu J., Wong W.T., Tian X.Y., Lau C.W., Wang Y.X., Xu G., Pu Y., Zhu Z., Xu A. (2012). Dipeptidyl peptidase 4 inhibitor sitagliptin protects endothelial function in hypertension through a glucagon-like peptide 1-dependent mechanism. Hypertension.

[49] Devin J.K., Pretorius M., Nian H., Yu C., Billings F., Brown N.J. (2014). Substance P increases sympathetic activity during combined angiotensin-converting enzyme and dipeptidyl peptidase-4 inhibition. Hypertension.

[50] Shah Z., Pineda C., Kampfrath T., Maiseyeu A., Ying Z., Racoma I., Deiuliis J., Xu X., Sun Q., Moffatt-Bruce S. (2011). Acute DPP-4 inhibition modulates vascular tone through GLP-1 independent pathways. Vasc. Pharmacol..

[51] Hwang H.J., Chung H.S., Jung T.W., Ryu J.Y., Hong H.C., Seo J.A., Kim S.G., Kim N.H., Choi K.M., Choi D.S. (2015). The dipeptidyl peptidase-IV inhibitor inhibits the expression of vascular adhesion molecules and inflammatory cytokines in HUVECs via Akt- and AMPK-dependent mechanisms. Mol. Cell Endocrinol..

[52] Marney A., Kunchakarra S., Byrne L., Brown N.J. (2010). Interactive hemodynamic effects of dipeptidyl peptidase-IV inhibition and angiotensin-converting enzyme inhibition in humans. Hypertension.

[53] De Oliveira T.V., Guimaraes A.P., Bressan G.C., Maia E.R., Coimbra J.S.D., Polêto M.D., De Oliveira E.B. (2021). Structural and molecular bases of angiotensin-converting enzyme inhibition by bovine casein-derived peptides: An molecular dynamics approach. J. Biomol. Struct. Dyn..

[54] Macip G., Garcia-Segura P., Mestres-Truyol J., Saldivar-Espinoza B., Ojeda-Montes M.J., Gimeno A., Cereto-Massagué A., Garcia-Vallvé S., Pujadas G. (2022). Haste makes waste: A critical review of docking-based virtual screening in drug repurposing for SARS-CoV-2 main protease (M-pro) inhibition. Med. Res. Rev..

[55] Megantara S., Rusdin A., Budiman A., Puluhulawa L.E., Ikram N.K.B.K., Muchtaridi M. (2025). Demonstrating the Absence of Correlation Between Molecular Docking and in vitro Cytotoxicity in Anti-Breast Cancer Research: Root Causes and Practical Resolutions. Breast Cancer.

