# Supplementary material for: Novel Dual DPP-IV/ACE Inhibitory Peptides from Tartary Buckwheat: From Inhibition Kinetics to Cellular Bioactivity
Source: Foods. 2026 Oct 31;15(21):3912. doi: 10.3390/foods15213912 (PMC13763512; doi:10.3390/foods15213912)
Supplement: Supplementary file 1 [file foods-15-03912-s001.zip › foods-4595435-supplementary.pdf]

Table S1. **Integrated virtual screening profile of candidate peptides from tartary buckwheat.**

No.	Peptide Sequence	MW (Da)	DPP-IV Prediction		ACE Prediction		GRAVY	pI	Safety Profile
			BIOPEP	StackDPPIV	BIOPEP	AHTpin (SVM)			Toxic/Allergenic
1	**VPIY**	492.60	0.50	0.700	0.50	1.91	1.450	5.81	Non/Non
2	Seq1	442.58	0.75	0.980	0.25	1.00	1.575	5.92	Non/Non
3	Seq2	340.40	0.67	0.970	0.33	-	2.233	5.75	Non/Non
4	Seq3	567.65	0.83	0.960	0.67	0.13	1.617	5.59	Non/Non
5	**IPLIGF**	657.77	0.67	0.700	0.67	0.52	2.267	5.51	Non/Non
6	Seq4	623.77	0.80	0.830	0.80	0.19	1.500	5.86	Non/Non
7	Seq5	570.75	0.80	0.710	0.60	0.92	0.120	6.22	Non/Non
8	Seq6	581.72	0.60	0.770	0.40	0.34	1.400	4.04	Non/Non
9	Seq7	552.68	0.80	0.660	0.20	0.13	2.540	5.77	Non/Non
10	Seq8	380.25	0.75	0.990	0.50	0.91	2.225	5.85	Non/Non
11	Seq9	464.57	0.75	1.000	0.25	0.81	1.600	5.80	Non/Non
12	Seq10	464.57	0.50	1.000	0.25	0.81	1.600	5.84	Non/Non
13	Seq11	426.56	0.50	0.850	0.50	1.09	2.475	5.77	Non/Non
14	Seq12	508.63	0.75	0.970	0.25	1.15	2.650	5.77	Non/Non
15	Seq13	772.86	0.67	0.940	0.50	-0.54	1.600	5.84	Non/Non
16	Seq14	776.89	0.67	0.700	0.67	-0.05	1.600	5.80	Non/Non

Note: GRAVY: Grand Average of Hydropathy; pI: Isoelectric point. Safety profiles were predicted using ToxinPred and AllerCatPro 2.0.

Table S2. **Inhibition constants (*K_i*) and α values from global nonlinear mixed-model fitting for VPIY and IPLIGF against DPP-IV and ACE.**

Peptide	Enzyme	α (95% CI)	K_i (μM, 95% CI)	Inhibition type
VPIY	DPP-IV	2.84 (1.72–5.12)	534.9 (443–661)	Mixed (α>1, CI excludes 1)
IPLIGF	DPP-IV	1.43 (0.86–2.42)	472.4 (367–642)	Non-competitive (α≈1, CI includes 1)
VPIY	ACE	1.10 (0.72–1.68)	144.3 (113–194)	Non-competitive (α≈1, CI includes 1)
IPLIGF	ACE	6.84 (3.90–15.7)	131.0 (111–156)	Competitive-like mixed (α≫1, CI excludes 1)

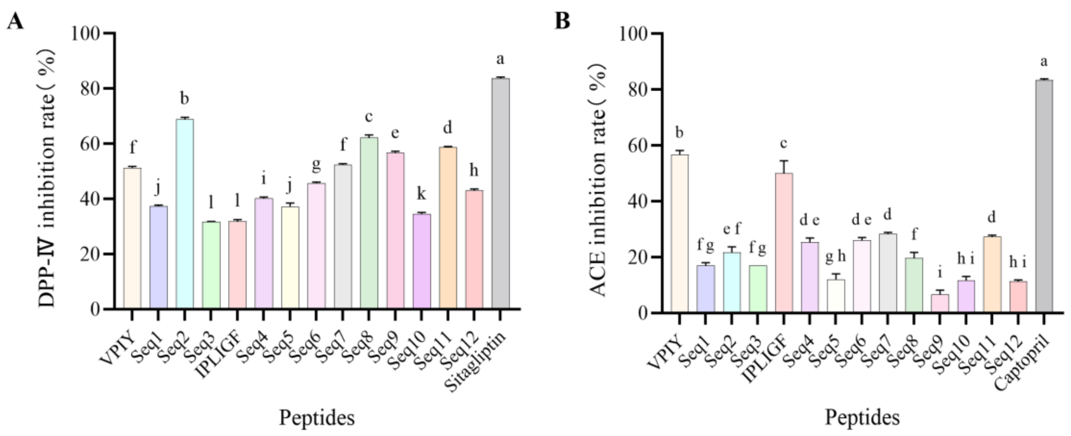

Figure S1. Initial screening of DPP-IV and ACE inhibitory activities of oligopeptides. (A) DPP-IV inhibitory rate (%) and (B) ACE inhibitory rate (%) of synthesized oligopeptides. Sitagliptin (1.19×10^{-5} M) and Captopril (1.43×10^{-5} M) were used as positive controls. Data are expressed as mean ± standard deviation (SD, n = 3). Bars with different lowercase letters (a–l) indicate significant differences among groups at $p < 0.05$ (one-way ANOVA followed by Tukey's test).
